# Altered Functional Integration in the Salience and Default Mode Networks in Euthymic Pediatric Bipolar Disorder

**DOI:** 10.1155/2020/5853701

**Published:** 2020-10-09

**Authors:** Weifang Cao, Haoran Chen, Qing Jiao, Dong Cui, Yongxin Guo, Weijia Gao, Jianfeng Qiu, Linyan Su, Guangming Lu

**Affiliations:** ^1^Department of Radiology, Shandong First Medical University & Shandong Academy of Medical Sciences, Tai'an 271016, China; ^2^Department of Radiology, Shandong Cancer Hospital and Institute, Shandong First Medical University and Shandong Academy of Medical Sciences, Jinan 250117, China; ^3^Department of Child Psychology, The Children's Hospital, Zhejiang University School of Medicine, Hangzhou 310003, China; ^4^Mental Health Institute of the Second Xiangya Hospital, Key Laboratory of Psychiatry and Mental Health of Hunan Province, Central South University, Changsha 410011, China; ^5^Department of Medical Imaging, Jinling Hospital, Clinical School of Medical College, Nanjing University, Nanjing 210002, China

## Abstract

Accumulating studies demonstrate emotional and cognitive dysregulation in the euthymic period of pediatric bipolar disorder (PBD). However, the relative contribution of functional integration in human brain to disturbed emotion and cognitive function in the euthymic PBD patients remains unclear. In this study, 16 euthymic PBD patients and 16 healthy controls underwent resting-state functional magnetic resonance imaging. A data-driven functional connectivity analysis was used to investigate functional connectivity changes of the euthymic PBD. Compared with healthy controls, the euthymic PBD exhibited greater global functional connectivity density in the left anterior insula and lower global functional connectivity density in the right temporoparietal junction, the left angular gyrus, and the bilateral occipital lobule. A distant functional connectivity analysis demonstrated altered integration within the salience and default mode networks in euthymic PBD. Correlation analysis found that altered functional connectivity of the salience network was related to the reduced performance in the backward digit span test, and altered functional connectivity of the default mode network was related to the Young Mania Rating Scale in euthymic PBD patients. Our findings indicated that disturbed functional integration in salience and default mode networks might shed light on the physiopathology associated with emotional and cognitive dysregulation in PBD.

## 1. Introduction

Bipolar disorder (BD) is a chronic and debilitating mental illness and has been increasingly diagnosed in pediatric age children (G. Z. [1]). In addition, retrospective studies indicated that symptoms in 55-60% of adults with BD begin in childhood or adolescence [2]. Therefore, it is important to understand the developmental pathophysiology of BD by investigating pediatric bipolar disorder (PBD). Substantial evidence indicates that cognitive impairment and emotional lability are present not only in periods of acute mood symptoms but also in periods of euthymia in PBD ([3]; G. Z. [1, 4]). However, little is known about the neurocognitive mechanisms of the euthymic phase of PBD.

Functional magnetic resonance imaging (fMRI) has been broadly used to investigate euthymic PBD [5, 6]. Many studies have provided evidence for changes of brain function in BD, such as changes in the corticolimbic pathways during mood episode [7, 8]. Recently, intrinsic functional connectivity (FC) based on resting-state fMRI has been employed to reveal stable and reliable functional brain networks, such as the default mode network (DMN), and the salience network (SN), which are associated with cognition and emotion [9, 10]. Moreover, altered functional connectivity has been considered a potential biomarker for psychiatric disorders [11, 12]. Many imaging studies have demonstrated inconsistent functional changes in brain networks in different neural phenotypes in BD patients due to heterogeneity in the analysis methods used [5, 13]. However, intrinsic functional connectivity analysis provides a useful approach to investigate the neural architecture of euthymic PBD patients.

In the current study, we firstly evaluated the global functional connectivity feature by using an unbiased data-driven method. These features are obtained from global functional connectivity density (FCD), which may be more sensitive in detection of functional alterations of the distribution of brain hubs [14, 15]. Then, we assessed the distant functional connectivity of those brain regions in which we observed a significantly different global FCD in euthymic PBD patients. We predicted that the euthymic PBD patients would exhibit altered functional connectivity, and these changes may contribute to the neural physiopathology of PBD.

## 2. Methods

### 2.1. Participants

This study recruited 16 PBD patients during clinical remission from the child and adolescent psychiatric clinic of the Second Xiangya Hospital of Central South University (Changsha, P. R. China) from January to July, 2012. All patients were required to meet the Diagnostic and Statistical Manual for Mental Disorders, Fourth Edition (DSM-IV) criteria for BD with a current remission episode. The exclusion criteria included the diagnosis of bipolar disorder subtype or current mixed episode. Sixteen age- and sex-matched healthy control (HC) subjects were recruited through advertisements in local public schools. All participants were 10-17 years old, right-handed and of Han ethnicity. They could follow the instructions to keep still during MRI scanning. The additional exclusion criteria for all participants included the presence of major sensorimotor handicaps, history of neurological disorder, history of electroconvulsive therapy, lower score of full-scale intelligence quotient (IQ < 80), and standard fMRI contraindications (e.g., metallic implants, retractors or braces, and claustrophobia). The study was approved by the ethics committee of the Second Xiangya Hospital of Central South University. Written informed consent was obtained from the parents of all participants.

### 2.2. Clinical and Psychological Assessment

All patients were diagnosed independently by two experienced psychiatrists for Affective Disorders and Schizophrenia for School aged Children Present and Lifetime Versions (K-SADS-PL). Current mental states were assessed using the Young Mania Rating Scale (YMRS) and Mood and Feelings Questionnaire (MFQ). To evaluate the effects of PBD on cognitive function, psychological measurements for all participants included the Digit Span Test (DST), the Trail Marking Test (TMT), and the Stroop Color-Word Test (SCWT). The DST includes the forward digit span test (DST-F) and backward digit span test (DST-B) to evaluate attention and executive processes. The SCWT is used to assess the ability to inhibit cognitive interference and is composed of three parts. The participant is asked to name the color of a series of dots in SCWT-A, to name the color-ink words in SCWT-B, and to name the color of words whose meanings are different from ink colors in SCWT-C. The TMT is frequently used neuropsychological tests in clinical practice and is comprised of part A (TMT-A) and part B (TMT-B). The TMT-A includes numbered circles only, whereas the TMT-B contains more numbered circles and squares. The participant is asked to connect these numbers in order.

### 2.3. Image Acquisition and Preprocessing Analysis

All image data were acquired using a Siemens 3T Siemens Trio scanner (Siemens, Munich, Germany). To minimize head motion, we fixed the subjects' heads using foam pads. The axial T1-weighted anatomical images were acquired using a spoiled gradient recall sequence, generating 176 slices (repetition time (TR) = 2300 ms, echo time (TE) = 2.03 ms, flip angle (FA) = 9°, slice thickness = 1 mm, matrix size = 256 × 256, and field of view (FOV) = 256 mm × 256 mm). Resting-state functional images were collected using echo planar imaging (EPI) [TR = 2000 ms, TE = 30 ms, FA = 90°, FOV = 240 mm × 240 mm, matrix = 64 × 64, and slice thickness = 4 mm], generating 30 slices. The functional scanning lasted for 510 s, yielding a total of 255 volumes. During the resting-state scanning, the subjects were asked to lie with their eyes closed, not to fall asleep, and not to think of anything in particular. To ensure magnetic field stabilization, the first five volumes were discarded.

All image data were preprocessed using the Neuroscience Information Toolbox (NIT) [16]. The preprocessing procedure included slice time correction, realignment, and spatial normalization (3 × 3 × 3 mm^3^). We excluded subjects with exceeded head motion (more than 2 mm translation or 2° rotational movements). The linear regression analysis was utilized to remove several nuisance covariates from the time course of all brain voxels of the functional data. These covariates included 12 head motion parameters (six head parameters and its derivative), white matter signal, cerebrospinal fluid signal, and global signal. Then, the temporal passband filtering (0.01-0.08 Hz) was conducted on the time courses of functional data to remove low-frequency drift and to minimize high-frequency physiological noise.

### 2.4. Global Functional Connectivity Density Analysis

For each subject, the global FCD map was obtained using Pearson's linear correlation from individual preprocessed time course. The threshold of the correlation coefficient, Tc, was used to determine significant connection between two voxels. The global FCD value at a given voxel was defined as the number of voxels with significant connections in the whole brain with the given voxel. The global FCD map reflected the total number of functional connections per voxel.

We chose a dynamic threshold range from 0.4 to 0.8 in 0.05 steps, to obtain more reliable and robust findings. The total nine Tc thresholds were used in our study. To address variability in all global FCD across subjects, each individual global FCD map was normalized by dividing by the mean value across voxels in a given subject. For all FCD maps, we created nine normalized maps for the nine Tc thresholds for each subject. Spatial smooth with 6 mm Gaussian kernel of full-width half maximum (FWHM) was used to minimize the differences in the functional anatomy of the brain across subjects.

Group analyses of all global FCD maps were conducted using one-sample *t-*tests (*P* < 0.05, FDR corrected). For each global FCD map for each threshold, Tc, we conducted a two-sample *t*-test comparing the euthymic PBD and HC groups, controlling for the age, gender, and education years.

To identify robust differences of global FCD between the euthymic PBD and HC groups, we only included the clusters exhibiting a significant difference in at least 4 consecutive Tc values of FCD comparisons. Then, these clusters were used as regions of interest (ROIs) for the subsequent functional connectivity analyses.

### 2.5. Functional Connectivity Analysis

For the FC analysis, the normalized functional images were further processed using spatial smoothing using a 6 mm Gaussian kernel of FWHM. Then, the general regression analysis was performed to minimize the reflection of nuisance signals, as mentioned above. After filtering (0.01-0.08 Hz), FC analysis was performed by calculating the Pearson's correlation coefficients between the average time course of each ROI and that of each voxel in the whole brain. A Fisher *z*-transformation for correlation coefficients was applied. Therefore, individual *Z*-score FC maps were defined for each ROI and subject. One-sample *t*-tests were conducted within the euthymic PBD and HC groups. Then, we performed two-sample *t*-tests to detect the differences in the FC maps between the euthymic PBD and HC groups within the masks from the union set of the one-sample tests results of the FC maps (*P* < 0.05, FDR corrected) of the two groups, controlling for age, education years, and gender effects.

### 2.6. Correlation Analyses between Functional Connectivity and Clinical and Psychological Measurements

We used a partial correlation analysis to explore the relationship between clinical and psychological measurements and FC features including the global FCD of each ROI and the FC map of ROIs, controlling for the effects of age, education years, and gender.

## 3. Results

There was no significant difference in gender, age, education years, IQ, MFQ, TMT, and DST-F scores between the euthymic PBD and HC groups (*P* > 0.05). The YMRS, SCWT, and DST-B scores showed significant difference between the two groups (*P* < 0.05).  Table 1 shows these clinical data and neuropsychological measurement in detail.

### 3.1. Global Functional Connectivity Density Analysis

**Figure 1:**
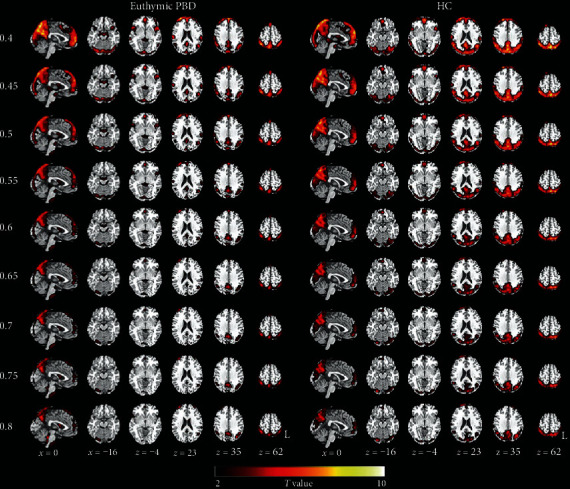
The global FCD maps for the nine Tc thresholds in the euthymic pediatric bipolar disorder (PBD) and healthy controls (HC) groups (one-sample *t*-test, *P* < 0.05, FDR corrected, cluster size > 621 mm^3^). L: left; R: right.

We illustrated the distribution of the global FCD for the nine Tc in the euthymic PBD and HC groups (Figure 1). For almost all Tc thresholds, we found the highest global FCD in the precuneus, angular gyrus, inferior parietal lobule, occipital cortex, superior temporal cortex, superior frontal gyrus, and the cerebellum, while the cluster size of the regions grew smaller as the Tc threshold increased. The pattern of regions exhibiting highest global FCD was similar as that of previous studies.

**Figure 2:**
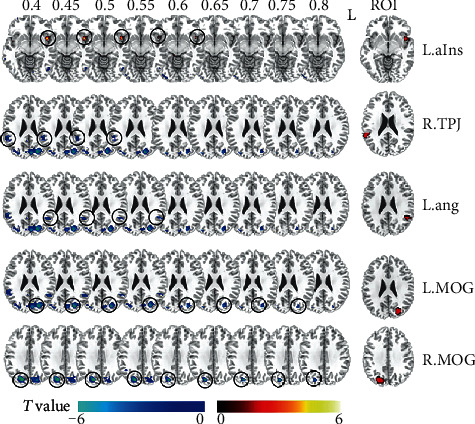
The differences of global FCD maps between the euthymic PBD and HC groups in 9 Tc thresholds separately (*P* < 0.005, cluster size > 270 mm^3^). The left part shows 5 ROIs' position and designation; L: left. ROIs' abbreviations are consistent with those shown in Table 2.

We conducted a two-sample *t*-test to compare data from the euthymic PBD group and HC group for each threshold (*P* < 0.005, cluster size > 270 mm^3^). We summarized all differences of global FCD resulting from the nine Tc thresholds to obtain stable differences between groups (Figure 2). Compared with the HC group, the euthymic PBD group showed increased global FCD in the left anterior insula at five consecutive Tc thresholds comparisons (from 0.4 to 0.6). We also found significantly decreased global FCD for more than half of thresholds at four clusters in the euthymic PBD group compared with the HC group. These clusters included the right angular gyrus, the left temporoparietal junction (TPJ), and the bilateral middle occipital cortex (MOG). In total, we identified five clusters of significantly altered global FCD in the euthymic PBD group. These regions were defined as regions of interest (ROIs) in the subsequent FC analysis (Table 2).

### 3.2. Functional Connectivity Analysis

**Figure 3:**
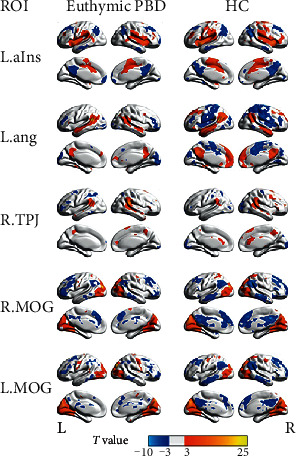
The group-level functional connectivity maps seeded at 5 ROIs in the euthymic pediatric bipolar disorder (PBD) and healthy control (HC) groups (one-sample *t*-test, *P* < 0.05, FDR corrected, cluster size > 621 mm^3^). L: left; R: right. ROIs' abbreviations are consistent with those shown in Table 2.

For each ROI, we assessed a whole brain FC map for each group using one-sample t-test (*P* < 0.05, FDR-corrected, cluster size > 621 mm^3^).  Figure 3 shows these connectivity patterns of the FC map in the euthymic PBD and HC groups. In both groups, the regions that showed a positive correlation with the left anterior insula included the bilateral insula, dorsal anterior cingulate cortex, bilateral TPJ, supplementary motor area, and bilateral middle prefrontal cortex, which had been identified as the salience network in previous studies. The right supramarginal gyrus showed positive correlation with bilateral supramarginal cortex, the right middle prefrontal cortex, the anterior cingulate cortex, and precuneus. The left angular, which was identified as a node of the default mode network, positively correlated with the precuneus, posterior cingulate cortex, bilateral ventromedial prefrontal and inferior parietal cortex, and bilateral superior temporal gyrus and crus II of the cerebellum. In two groups, the seeds of bilateral MOG positively correlated with the occipital cortex, cuneus lobe, calcarine gyrus, and lingual gyrus.

**Figure 4:**
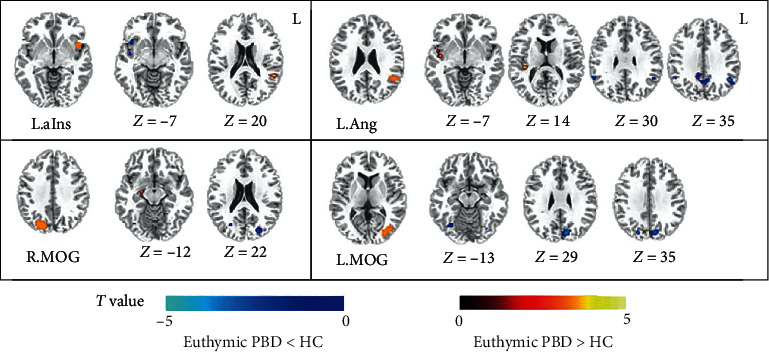
The difference of functional connectivity maps seeded at 4 ROIs between the euthymic pediatric bipolar disorder (PBD) and healthy control (HC) groups (*P* < 0.005, cluster size > 621 mm^3^). The left column for each subgraph shows the seed. The hot color represents higher correlation coefficients, and the cold color represents lower correlation coefficients in the euthymic pediatric bipolar disorder (PBD). ROIs' abbreviations are consistent with those shown in Table 2.

The results of the two-sample *t*-test showed that four of the five ROIs exhibited significant differences between the euthymic PBD and HC groups (*P* < 0.005, cluster size > 621 mm^3^) (Figure 4). Compared with the HC, the euthymic PBD exhibited decreased FC between the left anterior insula and right anterior insula and increased FC between the left anterior insula and the right TPJ. In the FC map of the left angular as seed, the euthymic PBD exhibited increased FC in the temporal gyrus and decreased FC in the bilateral angular and precuneus. The decreased FC between the bilateral MOG and the middle-inferior occipital cortex and cuneus were also identified in the euthymic PBD group (Table 3).

### 3.3. Results of Correlation Analyses

**Figure 5:**
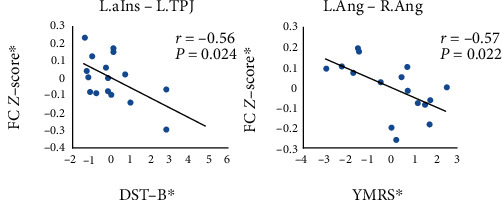
The relationship between the functional connectivity and psychological and clinical variables. ∗ represents the adjusted values controlling for the influence of the age, gender, and education years by the linear regression model.

The correlation analysis showed a negative relationship in the FC between the left anterior insula and the left TPJ and the DST-B (*r* = −0.559, *P* = 0.024) in the euthymic PBD group. The functional connectivity between the left angular and the right angular was negatively correlated to the YMRS score (*r* = −0.57, *P* = 0.022) in the euthymic PBD group (Figure 5).

## 4. Discussion

In this study, we demonstrated altered intrinsic FC in the brain of euthymic PBD using resting-state fMRI. We observed increased global FCD in the left anterior insula and decreased global FCD in the right TPJ, the left angular and bilateral occipital lobule in the euthymic PBD patients. Functional connectivity analyses based on ROI showed a distinct altered integration within the salience and default mode networks in euthymic PBD. Our findings indicate that resting-state FC based on a data-driven approach could be useful for evaluating altered pathophysiology in euthymic PBD patients.

Using global FCD analysis, we observed significantly increased global functional connectivity in the left insula and decreased global functional connectivity in the right TPJ in the euthymic PBD patients compared to controls. The anterior insula and TPJ have been identified as key regions involved in the salience network, which is implicated in monitoring, detecting, and regulating salient stimuli from the internal and external environment [17]. This integration of the salience network may play a role for fundamental cognitive and behavioral function, and its alteration was found to associate with alteration of psychiatric symptoms in psychosis [18, 19]. Consistent with previous researches, abnormal emotional processing, as well as cognitive impairment, such as attention, processing speed, and working memory, characterized euthymic PBD [4, 20]. In the study, we observed impaired neuropsychological functioning, including decreased scores of the CWSW and DST-B and more completion time of TMT-A in euthymic PBD patients compared to controls. These measurements are commonly employed to examine cognitive function, and successful completion of these tests requires reasonable allocation of attention, flexibility, and working memory [21, 22]. The insula is considered a critical hub in mediating dynamic interactions in oriented attention and internally self-related cognition [23]. The TPJ, which is located at the ventral-anterior section of the inferior parietal lobule and the posterior end of the superior temporal sulcus, is thought to either shift attention to goal-directed cognitive processing or understand others' mental state [24–26]. A recent task fMRI study had suggested that BD patients showed less activation and altered FC in regions within the salience network during an emotion-cognition integration task [7]. Our further voxel-based whole FC analysis showed enhanced FC between the left insula and the TPJ and decreased FC between the bilateral insula in the euthymic PBD patients. We found a negative correlation between the DST-B and FC between the left insula and the left TPJ in the euthymic PBD. Considering the critical role of the insula and TPJ in cognitive and emotional processes, our finding suggested that the altered functional integration within the salience network in euthymic PBD might contribute to the underlying mechanisms of impaired higher-level cognitive processes in patients.

Growing evidence has suggested that the DMN is compromised in many neuropsychiatric disorders [6, 27, 28]. The DMN, which contains the ventral-middle prefrontal cortex, bilateral angular gyrus, the precuneus (poster cingulate cortex), and bilateral temporal gyrus, is associated with self-referential and introspective states [29]. The left angular gyrus has been repeatedly implicated in episodic and semantic cognition, and the connection of the angular gyri with other DMN regions may act as a connector hub for global integration of information [30]. We observed the increased global FCD in the left angular gyrus in euthymic PBD patients. Further FC analysis revealed that the left angular gyrus showed increased FC with the right superior temporal cortex and decreased FC with the precuneus and right angular gyrus. Here, we observed a negative correlation between the FC of bilateral angular gyrus and the YMRS in the euthymic PBD. In other words, the lower FC between the bilateral angular gyrus was associated with the worse mental state. The precuneus involvement in introspective processes such as self-referential and emotional processing was associated with attenuated activity toward external events [29]. Therefore, we suggested that the altered interaction of regions of the DMN might serve to impair mental processing in the euthymic PBD patients.

The euthymic PBD patients exhibited decreased global FCD in the bilateral MOG, which may serve as the potential functional basis for the deficits in visual processing [31]. A few task fMRI studies found abnormalities in MOG during emotion face processing task in PBD patients [32, 33]. Our previous study also found decreased functional activity in MOG, which was associated with the performance of cognition in PBD patients (W. [34]). Therefore, we speculated that the reduced FC in the MOG might impair the perception during self-referential processing in the euthymic PBD patients.

Several limitations of our study must be noted. First, the sample size of participants was relatively small, and the larger samples would help to confirm results presented here. Second, our sample included the euthymic PBD patients with type I and type II. Although we agree that there might be differences in FC in subtypes of PBD, it would have been very difficult to exclude an entire subtype of BD according to our study design. Finally, the majority of PBD patients were taking psychotropic medication when scanning. Few studies have suggested that drug treatment may affect functional neuroimaging measures [35]. Future studies will use the prospective design to clarify the contribution of medicine and subtypes of PBD to altered neuroimaging features of PBD patients.

## 5. Conclusions

In summary, we demonstrated that the euthymic PBD exhibited increased global FCD in the left anterior insula and decreased global FCD in the left angular and bilateral occipital lobule. Functional connectivity analyses showed a distinct altered integration within the salience and default mode networks in euthymic PBD. The altered functional integration was associated with altered emotional and cognitive processing in the euthymic PBD. These findings indicate that disturbed functional integration in salience and default mode networks might have potential implications for the physiopathology of the PBD.

## Figures and Tables

**Table 1:** Comparison of clinical and neuropsychological measurement between the euthymic PBD patients and healthy controls.

	Euthymic PBD	HC	*P* value
Gender (male/female)	7/9	5/11	0.716^a^
Age (mean ± SD, years)	15.12 ± 1.71	14.06 ± 1.48	0.070^b^
Education (mean ± SD, years)	8.19 ± 1.80	7.19 ± 2.04	0.152^b^
Age of onset (mean ± SD, years)	13.13 ± 2.09		
BD type (I/II)	10/6		
Familial history (yes/no)	5/11		
Medications			
Lithium	6 (38%)		
Valproate	11 (69%)		
Atypical antipsychotics	13 (81%)		
K-SADS-PL comorbidity diagnoses			
ADHD	4 (25%)		
OCD	1 (6%)		
Anxiety	3 (19%)		
Tic	1 (6%)		
MFQ score (mean ± SD)	7.50 ± 4.37	5.56 ± 3.18	0.385^c^
IQ (mean ± SD)	106.69 ± 10.54	105.00 ± 7.00	0.609^c^
YMRS score (mean ± SD)	5.38 ± 1.69	3.59 ± 1.90	0.009^c^
SCWT-A (mean ± SD)	55.94 ± 11.34	64.81 ± 11.34	0.003^c^
SCWT-B (mean ± SD)	73.88 ± 13.41	86.56 ± 8.71	0.001^c^
SCWT-C (mean ± SD)	33.63 ± 7.54	39.50 ± 7.84	0.006^c^
TMT-A (mean ± SD)	37.83 ± 13.48	30.50 ± 9.29	0.004^c^
TMT-B (mean ± SD)	98.44 ± 49.70	82.17 ± 29.51	0.237^c^
DST-F (mean ± SD)	8.75 ± 1.81	9.00 ± 1.10	0.758^c^
DST-B (mean ± SD)	4.50 ± 1.41	5.87 ± 1.63	0.019^c^

^a^Chi-square test; ^b^two-sample *t*-test; ^c^ two-sample *t*-test controlled by age, gender, and education years. ADHD: attention-deficit/hyperactivity disorder; DST-B: backward digit span test; DST-F: forward digit span test; HC: healthy controls; IQ: intelligence quotient; K-SASADS-PL: Schedule for Affective Disorders and Schizophrenia for School-Age Children-Present and Lifetime Version; MFQ: Child Mood and Feelings Questionnaire; OCD: obsessive-compulsive disorder; PBD: pediatric bipolar disorder; SCWT: Stroop Color-Word Test; TMT-A: part A of trail making test; TMT-B: part B of trail making test; YMRS: Young Mania Rating Scale.

**Table 2:** Five ROIs determined in global FCD analysis.

Brain regions	Abbreviation	Peak MNI coordinate			Brodmann	Size of ROIs (mm^3^)
		*x*	*y*	*z*		
Left anterior insula	L.aIns	-45	9	-9	48	459
Right temporoparietal junction	R.TPJ	51	-39	24	42	405
Left angular	L.Ang	-45	-51	21	39	918
Left middle occipital gyrus	L.MOG	-24	-84	21	18, 19	3051
Right middle occipital gyrus	R.MOG	21	-81	33	18, 19	324

FCD: functional connectivity density; MNI: Montreal Neurological Institute; ROI: regions of interest.

**Table 3:** The significantly altered functional connectivity between the euthymic PBD and HC groups in 4 seed maps.

ROI	Regions	MNI coordinate			*T* value	Cluster size (mm^3^)
		*x*	*y*	*z*		
L.aIns	Right insula	42	15	-12	-3.51	648
	Left temporoparietal junction	-48	-45	1	4.61	1080
L.Ang	Right superior temporal cortex	37	-30	14	5.62	1674
	Left angular	-51	-54	33	-4.37	1215
	Right angular	56	-51	26	-4.15	702
	Precuneus	3	-59	33	-4.17	1566
L.MOG	Left cuneus	-9	-84	24	-4.78	3078
	Right inferior occipital cortex	39	-75	-9	-3.99	1971
R.MOG	Right hippocampus	24	-12	-15	5.57	648
	Left cuneus	-24	-84	21	-4.78	3024

ROIs' abbreviations are consistent with those shown in Table 2. HC: healthy controls; MNI: Montreal Neurological Institute; PBD: pediatric bipolar disorder; ROI: regions of interest.

## Data Availability

The fMRI data used to support the findings of this study are available from the corresponding author upon request.
